# Recent advances in neural mechanism of general anesthesia induced unconsciousness: insights from optogenetics and chemogenetics

**DOI:** 10.3389/fphar.2024.1360864

**Published:** 2024-04-09

**Authors:** Hui Gao, Jingyi Wang, Rui Zhang, Tao Luo

**Affiliations:** ^1^ School of Anesthesiology, Shandong Second Medical University, Weifang, China; ^2^ Department of Anesthesiology, Peking University Shenzhen Hospital, Shenzhen, China

**Keywords:** optogenetics, chemogenetics, general anesthesia, wakefulness, loss of righting reflex, neural circuits

## Abstract

For over 170 years, general anesthesia has played a crucial role in clinical practice, yet a comprehensive understanding of the neural mechanisms underlying the induction of unconsciousness by general anesthetics remains elusive. Ongoing research into these mechanisms primarily centers around the brain nuclei and neural circuits associated with sleep-wake. In this context, two sophisticated methodologies, optogenetics and chemogenetics, have emerged as vital tools for recording and modulating the activity of specific neuronal populations or circuits within distinct brain regions. Recent advancements have successfully employed these techniques to investigate the impact of general anesthesia on various brain nuclei and neural pathways. This paper provides an in-depth examination of the use of optogenetic and chemogenetic methodologies in studying the effects of general anesthesia on specific brain nuclei and pathways. Additionally, it discusses in depth the advantages and limitations of these two methodologies, as well as the issues that must be considered for scientific research applications. By shedding light on these facets, this paper serves as a valuable reference for furthering the accurate exploration of the neural mechanisms underlying general anesthesia. It aids researchers and clinicians in effectively evaluating the applicability of these techniques in advancing scientific research and clinical practice.

## 1 Introduction

General anesthesia is considered a state of reversible unconsciousness that is accompanied by amnesia, analgesia, and behavioral unresponsiveness, even to painful stimuli (Brown et al., 2010; Moody et al., 2021). With the advancement of general anesthesia technology, numerous individuals now undergo general anesthesia daily. Back in the 1980s, researchers initially unveiled that general anesthetics work by interacting with hydrophobic sites on proteins, and since then have made significant progress in identifying the molecular targets of general anesthetics (Melonakos et al., 2020). Nonetheless, in contrast to the exploration into the protein and molecular mechanisms of general anesthesia, our understanding of the neural circuit mechanism behind general anesthesia remains comparatively limited.

It is becoming increasingly evident that many of the neural circuits involved in the sleep–wake cycle are also relevant for general anesthesia. (Zhang et al., 2022; Espinosa-Juárez et al., 2023). Studying neural networks necessitates the utilization of advanced techniques for neural labeling and modulation, including optogenetics, chemogenetics, electrophysiology, calcium imaging, anterograde/retrograde tracing behavioral testing, and so on. Among these techniques, optogenetics and chemogenetics stand out as crucial and frequently employed methods in the investigation of neural circuits and functions because of their ability to specifically modulate the activity of neurons and neural circuits (Vlasov et al., 2018).

## 2 Optogenetics

The neuroanatomy of the brain is very complex, often with many different types of neurons concentrated in a single brain region, which makes it extremely difficult to accurately identify the function of a particular type of neuron using traditional research methods, such as electrical stimulation. However, optogenetics can specifically regulate the activity and function of target cells by expressing light-sensitive ion channels or opsins in certain neuron types (Melonakos et al., 2020).

Optogenetics can precisely regulate the temporal accuracy of specific neurons or neural circuits (Zhang et al., 2022). Opsins are usually introduced into neurons in the target region by injection of virus that contains the opsin gene, and experiments are performed approximately 3 weeks later. Activation or inhibition of neurons is achieved by targeting light to neurons through an optical fiber implanted in the brain to depolarize or hyperpolarize the cell membrane. Opsins are classified based on the range of wavelengths necessary to activate them. Through the use of brief light pulses, it is possible to precisely regulate neuronal firing on a millisecond time scale.

A variety of light-sensitive opsins are available, encompassing both excitatory opsins like channelrhodopsin 2 (ChR2), inhibitory opsins like halorhodopsin (NpHR) and archaerhodopsin (Arch). In order to improve the sensitivity of opsin to light pulses, thereby better aligning with scientific demands, the researchers further developed a series of variants. For instance, ChR2 variants ChETAH family (Iwai et al., 2011; Mattis et al., 2012) and ChIEF (Lin et al., 2009) can elicit ultra-fast firing frequencies (up to 200 Hz or more) in fast-spiking neurons (Gunaydin et al., 2010). Notably, a double mutant of ChR2 (Asp156Ala and Cys128Ser), referred to as the stabilized step-function opsin (SSFO)48 exhibits enhanced photosensitivity, accelerated depolarization kinetics and prolonged depolarization maintenance, up to 30 min (Berndt et al., 2009; Yizhar et al., 2011; Mattis et al., 2012).

## 3 Chemogenetics

Similar to optogenetics, chemogenetics is a technique that uses chemical molecules instead of light to manipulate the activity of neurons in specific cells temporally and reversibly. The technique works by introducing receptors that can be activated by the engineered ligand into neurons in the target brain region to regulate neuronal activity. These receptors can be activated by particular synthetic ecto-ligands, which are otherwise inert (Vlasov et al., 2018). Various protein classes have been engineered using this technology, including G protein-coupled receptors (Alexander et al., 2009), ligand-gated ion channels (Lerchner et al., 2007; Magnus et al., 2011), kinases (Chen et al., 2005; Cohen et al., 2005), and non-kinase enzymes (Redfern et al., 1999; Häring and Distefano, 2001; Armbruster et al., 2007; Vardy et al., 2015).

The primary chemogenetic tool widely employed is the Designer Receptors Exclusively Activated by Designer Drugs (DREADDs) (Armbruster et al., 2007). DREADDs are modified versions of G-protein-coupled receptors, originating from human toxin-base receptors.

They exhibit a low affinity for the natural ligand acetylcholine, but their affinity for synthetic ligands like clozapine-n-oxide (CNO) is significantly elevated (Armbruster et al., 2007; Roth, 2016). Among them, hM3Dq and hM4Di were the most commonly used excitatory and inhibitory DREADDs, respectively. Through the binding of CNO, hM3Dq stimulates neurons by augmenting intracellular calcium levels, while hM4Di decreases adenylate cyclase levels, thereby inhibiting neuronal activity (Vardy et al., 2015). In addition to this, a novel inhibitory DREADD based on the kappa-opioid receptor (KORD) has been recently discovered. KORD is activated specifically by salvinorin B, and is resistant to activation by endogenous opioid peptides. This provides the advantage of being able to activate and inhibit the same population of neurons using different ligands within the same animal model (Vardy et al., 2015).

One of the most appealing aspects of chemogenetics, in contrast to optogenetics, is its capacity for systemic delivery of engineered ligands. This eliminates the need for invasive intracranial fiber implantation (Poth et al., 2021). Second, a single dose is enough to provide lasting effects for up to several hours. Neural activation or inhibition can be induced in several brain regions without fear of damage to brain tissue or misinterpretation of results due to the thermal effects of light. Finally, in comparison to optogenetics, chemogenetics offers the advantage of not relying on laser equipment. This characteristic simplifies the operational process, making it relatively straightforward and user-friendly (Vlasov et al., 2018).

## 4 Neural circuits of general anesthesia

The most prominent characteristic of general anesthesia is reversible loss of consciousness. In nature, the existence of reversible changes in consciousness is limited to just two approaches: natural sleep and general anesthesia. There are many similar neurophysiological features between the two, including similar behavioral changes, autonomic nervous system responses, and electroencephalogram (EEG) patterns (both exhibiting slow wave activity and delta oscillations, 0.1–4 Hz) in addition to reversible changes in consciousness (Yang et al., 2022). Because of their similarity, researchers have grown increasingly intrigued by unraveling the underlying the neural circuitry governing general anesthesia and sleep-awake, along with their intricate interplay. In recent years, the rapid advancements in optogenetics and chemogenetics have provided invaluable tools for investigating brain function and deciphering neural circuit mechanisms.

**FIGURE 1:**
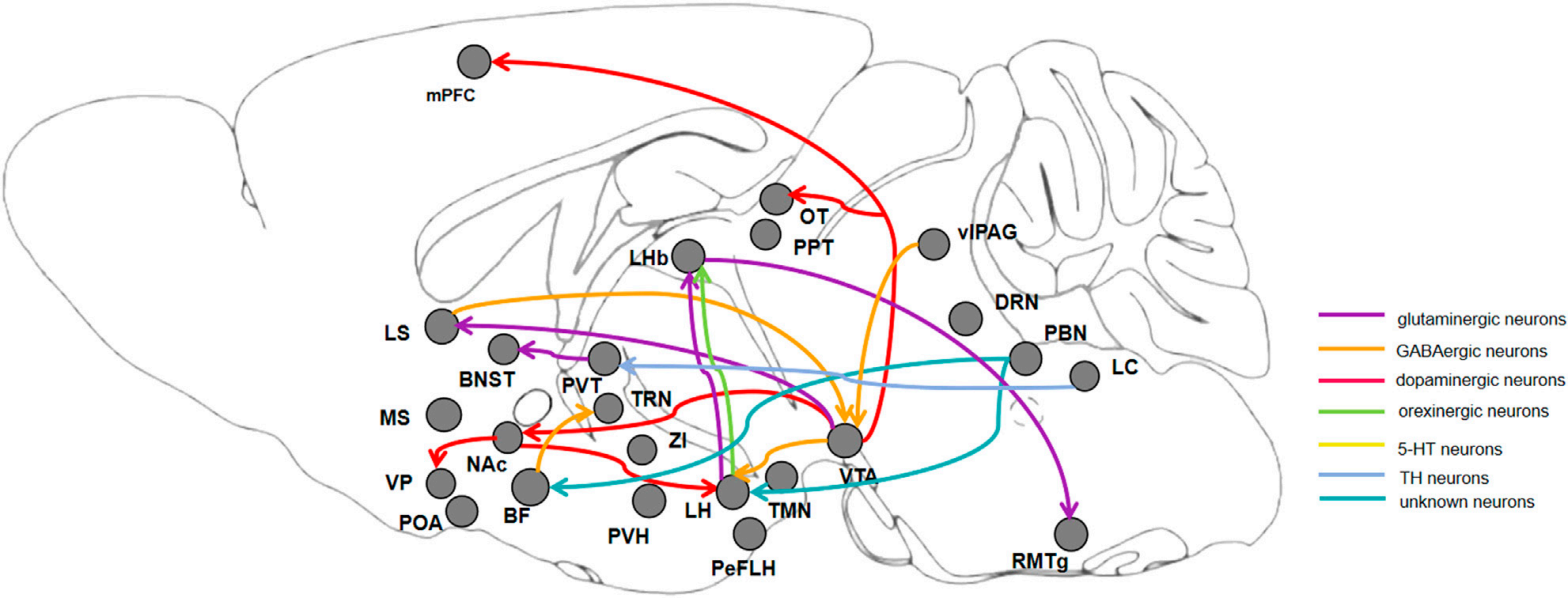
Diagram of connections between brain regions, showing neurons and neuronal circuits that regulate the progress of induction and emergence during general anesthesia. BF: basal forebrain; NAc: nucleus accumbens; LS: lateral septum; MS: medial septum; VTA: ventral tegmental area; PVT: paraventricular thalamus; TRN: thalamic reticular nucleus; ZI: zona incerta; LH: lateral hypothalamus; PVH: paraventricular hypothalamic nucleus; POA: preoptic area; TMN: hypothalamic tuberomammillary nucleus; PBN: parabrachial nucleus; LHb: lateral habenula; vlPAG: ventrolateral periaqueductal gray; DRN: dorsal raphe nucleus; LC: locus coeruleus; RMTg: rostromedial tegmental nucleus; PPT: pedunculopontine tegmental nucleus.

The methods used in these studies included behavioral and electroencephalogram (EEG) tests. The behavioral tests mainly include general anesthesia induction time, emergence time as well anesthetic concentration that induced loss of righting reflex. EEG can reflect the overall electrical activity of the animal’s brain and have been used for the prediction of the depth of anesthesia. EEG frequency bands is divided to delta, 0.5–4.0 Hz; theta, 4.0–9 Hz; alpha, 7–15 Hz; beta, 8–30 Hz; gamma, 40–300 Hz. Active or motivated wakefulness is rich in theta and gamma bands, whereas quiet wakefulness is characterized by slower EEG frequencies, including alpha and beta bands (Eban-Rothschild et al., 2018). As the depth of general anesthesia deepens, the EEG frequency slows down, such as the decrease of alpha wave and beta wave, the increase of delta wave and theta wave, etc., and the amplitude of the wave increases simultaneously (Jung and Kim, 2022). At deeper anesthesia, the EEG showed intermittent high power range oscillations (bursts) in alternation with isoelectricity (suppression), the so-called burst suppression state (Ching et al., 2012). Calculation of relative changes in total power by spectral analysis can reveal differences in the state of brain activity. In this context, we mainly reviewed studies employing optogenetics (Table 1) and chemogenetics (Table 2) to investigate general anesthesia-regulated nuclei and neural mechanisms intricately entwined within the sleep-wake neural circuits. The neural connections between brain regions that modulate general anesthesia are showed in Figure 1.

**TABLE 1:** Main findings of manipulating neural nuclei and circuits under general anesthesia with optogenetics.

Neuron type of brain region and its projections	Injection site	Place of Stimulation (light)	Experimental animals	Anesthetic method	activation (+)/inhibition (−)	Induction time	Emergence time	EEG recording	References
NAc D1R neurons and NAc D1R projections in the VP	NAc	NAc (473 nm, 30 Hz, 5 ms)	D1R-Cre mice	1.4% sevoflurane	+	——	——	power changes	Bao et al. (2021)
				2.0% sevoflurane	+	——	——	power changes, BSR increased	
		NAc (473 nm, 20 Hz, 10 ms during the LORR period and 589 nm, 10 Hz, 10 ms during the RORR period)		2.4% sevoflurane	+	prolonged by 14%	shortened by 34%	power changes, BSR decreased	Zhang et al. (2023)
		VP (473 nm, 20 Hz, 10 ms during the LORR period and 589 nm, 10 Hz, 10 ms during the RORR period)			+	no significance	shortened by 31%	power changes, BSR decreased	
					-	no significance	prolonged by 47%	power changes	
		VP (473 nm, 30 Hz, 5 ms)		1.4% sevoflurane	+	——	——	power changes	Bao et al. (2023)
		VP (473 nm, 30 Hz, 5 ms)		2.0% sevoflurane	+	——	——	power changes, BSR decreased	
		LH (473 nm, 30 Hz, 5 ms)		1.4% sevoflurane	+	——	——	power changes	
		LH (473 nm, 30 Hz, 5 ms)		2.0% sevoflurane	+	——	——	power changes, BSR decreased	
NAc D2R neurons and NAc D2R projections in the VP	NAc	NAc (473 nm, 20 Hz, 10 ms)	D2R-Cre mice	2.4% sevoflurane	+	shortened by 20%	prolonged by 25%	power changes	Niu et al. (2023)
		NAc (589 nm, 20 Hz, 10 ms)			-	prolonged by 18%	shortened by 20%	power changes	
		VP (473 nm, 20 Hz, 10 ms)			+	shortened by 22%	no significance	power changes	
		VP (589 nm, 20 Hz, 10 ms)			-	prolonged by 25%	no significance	power changes	
NAc GABAergic neurons	NAc	NAc (473 nm, 20 Hz, 10 ms)	Vgat-Cre mice	propofol, 150 mg/kg	+	prolonged by 20%	shortened by 31%	power changes	Yan et al. (2023)
		NAc (594 nm, 20 Hz, 10 ms)			-	shortened by 34%	prolonged by 45%	power changes	
LS GABAergic neurons	LS	LS (473 nm, 20 Hz, 10 ms)	Vgat-Cre mice	1.5% isoflurane	+	——	shortened by 56%	power changes, BSR decreased	Wang et al. (2021a)
		LS (589 nm, 20 Hz, 10 ms)			-	——	prolonged by 94%	power changes	
		VTA (473 nm, 20 Hz, 10 ms)			+	——	shortened by 68%	power changes	
		VTA (589 nm, 20 Hz, 10 ms)			-	——	prolonged by 91%	power changes	
MS glutamatergic neurons	MS	MS (473 nm, 10 Hz, 5 ms)	Vglut2-Cre mice	1.8% sevoflurane	+	——	——	power changes	Xia et al. (2024)
				2% sevoflurane	+	——	——	BSR decreased	
BF cholinergic neurons and glutamatergic neurons	BF	BF (473 nm, 20 Hz, 10 ms)	ChAT mice, wild type mice	propofol, 200 mg/kg	+	prolonged by 77%	shortened by 50%	power changes	Wang et al. (2021b)
			C57BL/6 mice		+	prolonged by 64%	shortened by 27%	power changes	
BF GABAergic neurons	BF	BF (blue light 20 Hz, 10 ms)	Vgat-Cre mice	1.4% isoflurane	+	prolonged by 37%	shortened by 79%	BSR decreased	Cai et al. (2023)
		TRN (blue light 20 Hz, 10 ms)		1.4% isoflurane	+	prolonged by 25%	shortened by 61%	BSR decreased	
		BF or TRN (blue light 20 Hz, 10 ms)		0.8% isoflurane	+	——	——	power changes	
VTA DA neurons and VTA-PrL and VTA-NAc DA projections	VTA	VTA (473 nm, 30 Hz, 10 ms)	DAT-cre mice	0.8%-0.9%isoflurane	+	——	——	power changes	Taylor et al. (2016)
		PrL (473 nm, 25 Hz, 20 ms)	SD rats	2.4% sevoflurane	+	prolonged by 45%	shortened by 87%	power changes	Song et al. (2022)
		VTA (473 nm, 20 Hz, 15 ms)	DAT-cre mice	2.4% sevoflurane	+	prolonged by 12%	shortened by 18%	power changes	Gui et al. (2021)
		NAc (473 nm, 20 Hz, 15 ms)			+	prolonged by 21%	shortened 15%	power changes	
		NAc (589 nm, 10 Hz, 20 ms)			-	reduced by 20%	prolonged by 43%	power changes	
		OT (473 nm, 20 Hz, 10 ms)	C57BL/6 mice	1.4% isoflurane	+	no significance	shortened by 37%	power changes	Yang et al. (2021)
VTA GABAergic neurons and projections in LH	VTA	VTA (473 nm, 20 Hz, 30 ms)	Vgat-Cre mice	1.0% isoflurane	+	——	——	power changes, BSR increased	Yin et al. (2019)
		VTA (594 nm, 1 Hz, 1s)			-	——	——	power changes, BSR decreased	
		LH (473 nm, 20 Hz, 30 ms)		1.4% isoflurane	+	shortened by 30%	prolonged by 43%	power changes, BSR increased	
		LH (594 nm, 1 Hz, 1s)			-	prolonged by 44%	no significance	power changes, BSR decreased	
VTA glutamatergic neurons and projections in LS	VTA	VTA (473 nm, 20 Hz, 20 ms)	vGlut2-Cre mice	1.4% isoflurane	+	prolonged by 36%	shortened by 24%	BSR decreased	Zhang et al. (2023b)
				0.8% isoflurane	+	——	——	power changes	
		VTA (594 nm, 1 Hz, 1s)		1.4% isoflurane	-	shortened by 58%	prolonged by 12%	BSR increased	
				0.8% isoflurane	-	——	——	power changes	
		LS (473 nm, 20 Hz, 20 ms)		1.4% isoflurane	+	prolonged by 21%	shortened by 15%	BSR decreased	
				0.8% isoflurane	+	——	——	power changes	
		LS (594 nm, 1 Hz, 1s)		1.4% isoflurane	-	shortened by 14%	prolonged by 13%	BSR increased	
				0.8% isoflurane	-	——	——	power changes	
PVT glutamatergic neurons and BNST terminalis	PVT	PVT (473 nm, 10 Hz,10 ms)	vGlut2-Cre mice	1.5% isoflurane	+	——	shortened by 38%	——	Ren et al. (2018b)
		BNST (473 nm, 10 Hz, 10 ms)	vGlut2-Cre mice	2.4% sevoflurane	+	prolonged by 50%	shortened by 35%	power changes, BSR decreased	Li et al. (2022)
PVT calretinin-expressing neurones	PVT	PVT (blue light 30 Hz, 5 ms)	CR-Cre mice	propofol, 3 mg/kg/min	+	——	——	power changes	Wang et al. (2023b)
		PVT (blue light 20 Hz, 5 ms or yellow light 20 Hz,5 ms)		propofol, 10 mg/kg/min	-	shortened by 23%	prolonged by 61%	no significance	
TRN GABAergic neurons	TRN	TRN (473 nm, 20 Hz, 30 ms)	Vgat-Cre mice	propofol, 20 mg/kg	+	no significance	shortered by 22%	——	Liu et al. (2021a)
		TRN (596 nm, 1Hz, 1s)			-	no significance	prolonged by 31%	——	
ZI GABAergic neurons	ZI	ZI (473 nm, 20 Hz, 20 ms)	C57BL/6 J mice	2% sevoflurane	+	shortened by 29%	no significance	——	Cao et al. (2023)
				2%propofol, 100 mg/kg	+	shortened by 28%	no significance	——	
LH glutamatergic neurons and LH-LHb projections	LH	LH (473 nm, 20 Hz, 30 ms)	vGlut2-Cre mice	0.8%/1.0% isoflurane	+	——	——	power changes, BSR decreased	Zhao et al. (2021a)
		LH (594 nm, 1 Hz, 1 s)			-	——	——	power changes	
		LHb (473 nm, 20 Hz, 30 ms)		1.4% isoflurane	+	prolonged by 13%	shortened by 39%	power changes, BSR decreased	
		LHb (594 nm, 1 Hz, 1 s)			-	shortened by 21%	prolonged by 50%	power changes	
LH orexinergic neurons and orexinergic terminals in the BF, LC, PVT and LHb	LH	LH (473 nm, 20 Hz, 20 ms)	orexin-Cre mice	2% isoflurane	+	——	shortened by 54%	——	Xiang et al. (2022)
	LH	LHb (473 nm, 20 Hz, 30 ms)	Hcrt-cre mice	2.4% sevoflurane	+	——	——	BSR decreased	Zhou et al. (2023)
				1.4%–1.7% sevoflurane	+	——	——	power changes	
		LHb (594 nm, 1 Hz, 1 s)		2.4% sevoflurane	-	——	——	BSR increased	
	PeFLH	BF (473 nm, 20 Hz, 10 ms)	Hcrt-cre rats	1.4% isoflurane	+	——	shortened by 28%	power changes, BSR decreased	Wang et al. (2021c)
		LC (473nm, 20 Hz, 10 ms)			+	——	shortened by 19%	power changes, BSR decreased	
	PeFLH	PeFLH (473 nm, 20 Hz, 30 ms)	Hcrt-Cre rats	7.6% desflurane anesthesia or 1.4% isoflurane	+	——	——	power changes, BSR decreased	Zhao et al. (2021b)
		PeFLH (594 nm, 1 Hz, 1 s)			-	——	——	power changes	
		PVT (473 nm, 20 Hz, 30 ms)			+	——	——	power changes, BSR decreased	
LH GABAergic projections to TRN	LH	TRN (473 nm, 20 Hz, 5 ms)	Vgat-Cre mice	1%–1.2% isoflurane	+	——	——	power changes, BSR decreased	Herrera et al. (2016)
TMN GABAergic neurons	TMN	TMN (473 nm, 20 Hz, 10 ms)	Vgat-Cre mice	2% sevoflurane	+	prolonged by 26%	shortened by 17%	power changes	Liu et al. (2023)
				propofol, 20 mg/kg	+	——	shortened by 7%	power changes	
PBN glutamatergic neurons	PBN	PBN (473 nm, 30 Hz, 5 ms)	vGlut2-Cre mice	2% sevoflurane	+	——	——	power changes	Wang et al. (2019)
PBN-LH projections and PBN-BF projections	PBN	LH (473 nm, 20 Hz, 15 ms)	C57BL/6 J mice	1.5% isoflurane	+	no significance	shortened by 22%	BSR decreased	Lu et al. (2023)
		LH (594 nm, 1 Hz, 1s)			-	no significance	prolonged by 31%	no significance	
		BF (473 nm, 30 Hz, 15 ms)			+	no significance	shortened by 13%	BSR decreased	
		BF (593 nm, 1 Hz, 1 s)			-	no significance	prolonged by 17%	no significance	
LHb glutamatergic neurons and LH-RMTg projections	LHb	LHb (473 nm, 10 Hz, 10 ms)	vGlut2-Cre mice	1.4% isoflurane anesthesia	+	shortened by 42%	prolonged by 35%	power changes	Liu et al. (2021b)
	LHb	RMTg (473 nm, 10 Hz, 10 ms)			+	shortened by 33%	prolonged by 15%	power changes	
vlPAG GABAergic neurons and vlPAG-VTA pathway and mPFC-vlPAG pathway	vlPAG	vlPAG (473 nm, 20 Hz, 10 ms)	Vgat-Cre mice	2% sevoflurane	+	no significance	shortened by 44%	BSR decreased	Guo et al. (2023)
		vlPAG (473 nm, 20 Hz, 10 ms)		1.5% sevoflurane	+	——	——	power changes	
		VTA (473 nm, 20 Hz, 10 ms)		2% sevoflurane	+	no significance	shortened by 26%	power changes, BSR decreased	
		VTA (473 nm, 20 Hz, 10 ms)		1.5% sevoflurane	+	——	——	power changes	
	mPFC	vlPAG (635 nm, 20 Hz, 10 ms)		2% sevoflurane	+	no significance	shortened by 29%	BSR decreased	
DRN 5-HT neurons	DRN	DRN (473 nm, 20 Hz, 20 ms)	Sert-Cre mice	1.0% isoflurane	+	——	——	BSR decreased	Li et al. (2021)
		DRN (594 nm,1 Hz, 1 s)			-	——	——	BSR increased	
		DRN (465 nm, 20 Hz, 20 ms)	C57BL/6 J mice	sevoflurane, induced at 8%, maintained at 2%	+	no significance	shortened by 49%	——	Ma et al. (2023)
LC TH neurons and LC-PVT projections	LC	LC (473 nm, 20 Hz, 10 ms)	TH-cre mice	0.8% isoflurane	+	——	——	power changes	Ao et al. (2021)
		PVT (473nm, 10 Hz, 10 ms)		1.2% isoflurane	+	no significance	shortened by 30%	power changes	
PPT glutamatergic neurons and PPT-VTA pathway	PPT	PPT (blue light, 30 Hz, 5 ms)	vGlut2-Cre mice	1.4%–1.5% sevoflurane	+	——	——	power changes	Li et al. (2024)
		PPT (blue light, 30 Hz, 5 ms)		2.5% sevoflurane	+	——	——	BSR decreased	
		VTA (blue light, 30 Hz, 5 ms)		2.5% sevoflurane	+	——	——	BSR decreased	

**TABLE 2:** Main findings of manipulating neural nuclei and circuits under general anesthesia with chemogenetics.

Neuron type of brain region and its projections	Place of stimulation	Experimental animals	Neurons activation (+)/inhibition (−)	ED50: increase (↑)or decrease (↓)	Anesthetic method	Induction time	Emergence time	References
NAc D1R neurons	NAc	D1R-Cre mice	+	↑	2.0% sevoflurane	prolonged by 32%	shortened by 36%	Bao et al. (2021)
			-	↓		shortened by 32%	prolonged by 177%	
NAc GABAergic neurons	NAc	Vgat-Cre mice	+	↑	propofol, 150 mg/kg	prolonged by 32%	shortened by 28%	Yan et al. (2023)
			-	↓		shortened by 46%	prolonged by 39%	
BF GABAergic neurons	BF	Vgat-Cre mice	+	↑	1.4% isoflurane	prolonged by 62%	shortened by 66%	Cai et al. (2023)
BF astrocytes	BF	Aldh1l1-creERT2 mice	+	——	1.4% isoflurane	shortened by 20%	prolonged by 43%	Lin et al. (2023)
VTA DA neurons and projections in PrL and NAc	VTA	SD rats	+	——	2.4% sevoflurane	prolonged by 30%	shortened by 35%	Song et al. (2022)
	PrL		+	——		prolonged by 24%	shortened by 31%	
	NAc	DAT-cre mice	+	——		prolonged by 20%	shortened by 21%	Gui et al. (2021)
			-	——		shortened by 19%	prolonged by 59%	
VTA GABAergic neurons	VTA	Vgat-Cre mice	+	↓	1.4% isoflurane	shortened by 25%	prolonged by 65%	Yin et al. (2019)
			-	↑		prolonged by 23%	shortened by 35%	
PVT glutamatergic neurons and BNST terminalis	PVT	C57BL/6J mice	-	↓	2.4% sevoflurane	shortened by 26%	prolonged by 63%	Li et al. (2022)
	BNST		-	↓		shortened by 36%	prolonged by 68%	
	PVT	vGlut2-Cre mice	+	——	1.4% isoflurane	no significance	shortened by 59%	Bu et al. (2022)
			-	——		no significance	prolonged by 59%	
	PVT	C57BL/6J mice	-	——	esketamine 5 mg/kg, 1.5% isoflurane	prolonged by 49%	prolonged by 30%	Duan et al. (2024)
			-	——	1.5% isoflurane	shortened by 21%	prolonged by 9%	
PVT calretinin-expressing neurones	PVT	CR-cre mice	+	↑	propofol, induced at 10 mg/kg/min for 5.5 min, maintained at 3.0 mg/kg/min for 30 min	prolonged by 18%	shortened by 43%	Wang et al. (2023b)
			-	↓		shortened by 28%	prolonged by 99%	
TRN GABAergic neurons	TRN	Vgat-Cre mice	+	——	propofol, 20 mg/kg	no significance	shortened by 20%	Liu et al. (2021a)
			-	——		no significance	prolonged by 19%	
LH glutamatergic neurons and orexinergic neurons and orexinergic terminals in the LHb	LH	vGlut2-Cre mice	+	——	1.4% isoflurane	prolonged by 13%	shortened by 35%	Zhao et al. (2021a)
			-	——		shortened by 19%	prolonged by 54%	
	LH	vGlut2-Cre mice	-	——	propofol, 10 mg/kg	prolonged by 33%	—	Huang et al. (2024)
			+	——		shortened by 30%	—	
	PeFLH	Hcrt cre rats	+	↑	7.6% desflurane or 1.4% isoflurane	prolonged by 19% (des.)	shortened by 15% (des.) or 27% (iso.)	Zhao et al. (2021b)
			-	↓		shortened by 21% (des.)	prolonged by 13% (des.) or 24% (iso.)	
	LHb	Hcrt cre mice	+	——	2.4% sevoflurane	no significance	no significance	Zhou et al. (2023)
			-	——		no significance	prolonged by 21%	
PVH glutamatergic neurons	PVH	vGlut2-Cre mice	+	——	1.0% isoflurane	prolonged by 20%	shortened by 36%	Yin et al. (2023)
			+	——	1.4% isoflurane	prolonged by 19%	shortened by 53%	
			-	——	1.0% isoflurane	shortened by 18%	prolonged by 33%	
			-	——	1.4% isoflurane	shortened by 21%	prolonged by 49%	
POA GABAergic neurons	MnPO or VLPO	Vgat-Cre mice or vGlut2-cre mice	+	——	1.5% or 1.2% isoflurane	no significance	no significance	Vanini et al. (2020)
			-	——		no significance	no significance	
TMN GABAergic neurons	TMN	Vgat-Cre mice	+	↑	2% sevoflurane	prolonged by 20%	shortened by 22%	Liu et al. (2023)
			-	↓		shortened by 12%	prolonged by 15%	
PBN glutamatergic neurons	PBN	vGlut2-Cre mice	+	↑	2% sevoflurane	prolonged by 56%	shortened by 73%	Wang et al. (2019)
			-	↓		no significance	prolonged by 55%	
LHb glutamatergic neurons	LHb	vGlut2-Cre mice	+	——	1.4% isoflurane	shortened by 40%	prolonged by 30%	Liu et al. (2021b)
			-	——		prolonged by 30%	shortened by 30%	
vlPAG GABAergic neurons	vlPAG	Vgat-Cre mice	+	——	2.0% sevoflurane	no significance	shortened by 20%	Guo et al. (2023)
			-	——		no significance	prolonged by 28%	
DRN serotonergic neurons	DRN	Sert-Cre mice	+	——	1.4% isoflurane	no significance	shortened by 18%	Li et al. (2021)
			-	——		no significance	prolonged by 30%	
LC TH neurons and LC-PVT projections	PVT	TH-cre mice	-	——	1.2% isoflurane	no significance	prolonged by 21%	Ao et al. (2021)
RMTg GABAergic neurons	RMTg	Vgat-Cre mice	+	↓	sevoflurane	——	——	Vlasov et al. (2021)
PPT glutamatergic neurones	PPT	vGlut2-Cre mice	-	↓	2.4% sevoflurane	shortenedby 37%	prolonged by 91%	Li et al. (2024)

## 5 Basal forebrain

The basal forebrain (BF) is a heterogenous region composed of multiple areas, including substantia innominate, nucleus accumbens and ventral pallidum of the basal ganglia, bed nucleus of the stria terminalis, the preoptic area, the nucleus basalis of Meynert, the septal nuclei and diagonal band of Broca (Tsanov, 2022). Cholinergic, glutamatergic and GABAergic neurons are the major neuronal populations in the basal forebrain (Xu et al., 2015). The basal forebrain is widely recognized for its crucial involvement in arousal, attention, reward processing, and various cognitive functions, as indicated by several studies (Voytko et al., 1994; Martinez et al., 2005; Fuller et al., 2011; Brown et al., 2012; Fischer et al., 2014; Anaclet et al., 2015; Agostinelli et al., 2019). Their role in general anesthesia has since been extensively studied.

### 5.1 Nucleus accumbens (NAc)

The nucleus accumbens (NAc) as the major structure of the ventral striatum, which participated in behaviors that are highly dependent on arousal, including locomotion, learning, impulsivity, risk-taking behaviors, feeding behavior, sexual motivation, as well as incentive and reward (Salgado and Kaplitt, 2015). Recent studies have illustrated that the nucleus accumbens is directly involved in sleep-wake regulation.

#### 5.1.1 Nucleus accumbens dopaminergic neurons

To investigate the role of dopaminergic neurons of the nucleus accumbens in general anesthesia, Bao et al. conducted the following study. Chemogenetic activation of the nucleus accumbens dopamine D1 receptor (D1R)-expressing neurons reduced sevoflurane sensitivity, prolonged the induction time of sevoflurane anesthesia. Additionally, optogenetic activation of these neurons induced significant cortical activation and behavioral emergence. Moreover, EEG analysis showed that optogenetic activation induced a decrease in delta waves and an increase in beta waves during continuous steady-state general anesthesia, and a significant reduction in burst-suppression ratio (BSR) during a deep anesthesia state. On the contrary, chemogenetic inhibition of these neurons increased sevoflurane sensitivity, accelerated induction and prolonged emergence from anesthesia (Bao et al., 2021). Similarly, Zhang et al. found that optogenetic activation of dopamine D1 receptor-expressing neurons in the nucleus accumbens prolonged the induction time, shortened the emergence time and resulted in an obvious decrease in BSR. Moreover, EEG recordings showed optogenetic activation of these neurons exhibited a decrease in delta and theta waves and an increase in beta waves during the induction period. In addition, during the emergence period, there was a decreased delta waves and an increased beta and gamma waves (Zhang J. et al., 2023).

The ventral pallidum (VP), is a component of the basal ganglia, located ventrally in the anterior fissure, with the primary source of innervation being the nucleus accumbens (Zhang J. et al., 2023). Optogenetic activation of the terminals of the nucleus accumbens dopamine D1 receptor-expressing neurons in the ventral pallidum also shortened the emergence time and reduced BSR. Additionally, EEG spectral analysis demonstrated that optogenetic activation induced a significant decrease in delta waves during the induction period, and an obvious decrease in delta band and an increase in beta and gamma bands during the emergence period. On the contrary, optogenetic inhibition of the terminals of the nucleus accumbens dopamine D1 receptor-expressing neurons in the ventral pallidum extended the emergence time. Furthermore, EEG spectral analysis showed that optogenetic inhibition induced an increase in beta waves during the induction period, and a significantly increased delta waves and a decreased alpha, beta and gamma waves during the emergence period (Zhang J. et al., 2023). Bao et al. also found that optogenetic activation of the terminals of the nucleus accumbens dopamine D1 receptor-expressing neurons in the ventral pallidum induced cortical activation and behavioral emergence (Bao et al., 2023).

Optogenetic activation of the terminals of the nucleus accumbens dopamine D1 receptor-expressing neurons in the lateral hypothalamus was also effective in promoting arousal after sevoflurane anesthesia. Under light anesthesia, brief blue-light stimulation of these neurons promoted activation of cortical EEG/EMG during sevoflurane maintenance, and optogenetic stimulation restored the righting reflex at a higher rate. Under deep anesthesia, optogenetic activation of these neurons changed the EEG pattern from the burst suppression to a relatively active state with low amplitude and high frequency waves. In addition, the BSR of ChR2 mice decreased significantly during optical stimulation (Bao et al., 2023). Overall, these results suggest that the nucleus accumbens dopamine D1 receptor-expressing neurons has a modulatory effect on the induction and emergence processes of sevoflurane anesthesia.

In addition, studies of other subtypes of the nucleus accumbens dopamine neurons led to opposite conclusions. Optogenetic activation of dopamine D2 receptor (D2R) expressing neurons located in the nucleus accumbens shortened the induction time and prolonged the emergence time. EEG spectral analysis revealed that optogenetic activation of these neurons during LORR led to an increase in delta bands and a decrease in gamma bands, and that during RORR led to an increase in delta bands and a decrease in beta and alpha bands. Conversely, optogenetic inhibition of the dopamine D2 receptor expressing neurons in the nucleus accumbens revealed that the neurons delayed sevoflurane induction and accelerated arousal, and EEG spectral analysis during LORR showed a decrease in the delta band and an increase in the delta, alpha and gamma bands, and a decrease in the delta and theta bands and an increase in the gamma band during RORR (Niu et al., 2023).

They further explored the role of the nucleus accumbens dopamine D2 receptor-expressing neurons in the ventral pallidum in regulating general anesthesia. Optogenetic activation of the nucleus accumbens dopamine D2 receptor-expressing neurons in the ventral pallidum accelerates sevoflurane induction, and during LORR resulted in delta bands increased, alpha, theta and gamma bands reduction. Optogenetic inhibition of this pathway prolonged the induction time, and during LORR resulted in delta bands reduction, alpha bands and gamma bands increase. while no significant effect was observed on the recovery of righting reflex time. These findings suggest that modulating the activity of this pathway can effectively regulate the induction process of sevoflurane anesthesia in mice (Niu et al., 2023). From the results of their experiments, it appears that the two subtypes of dopamine neurons in the nucleus accumbens play different roles in anesthesia. This outcome might arise from the distinct projection areas of these two neuron populations.

#### 5.1.2 Nucleus accumbens GABAergic neurons

Chemogenetic activation of the nucleus accumbens GABAergic neurons reduced propofol sensitivity, prolonged the induction time of propofol anesthesia, and promoted emergence from propofol anesthesia. Furthermore, EEG spectral analysis indicated that the delta power percentage was considerably lower than the control group during the induction and emergence periods. However, chemogentic inhibition of the nucleus accumbens GABAergic neurons had the opposite effect in both behavioral assessments and EEG spectral analysis. During the induction period, optogenetic activation of the nucleus accumbens GABAergic neurons resulted in a significant drop in delta power, while changes were minor during the induction period. Steady stimulation of the nucleus accumbens GABAergic neurons during the emergence period induced a clear decrease in delta power, whereas changes were small during the induction period. Notably, optogenetic inhibition of the nucleus accumbens GABAergic neurons revealed an obvious increase in delta power during the induction period and no difference during the emergence period. Furthermore, EEG data analysis showed that continuous stimulation during the emergence period induced a considerable increase in delta power, but changes were small during the induction period. Besides, behavioral results showed that after the stimulation, the emergence time was much longer but the induction time did not differ. These findings suggest that the nucleus accumbens GABAergic neurons regulate the progress of induction and emergence (Yan et al., 2023).

### 5.2 The septal nucleus

#### 5.2.1 Lateral septum

The septal nucleus can be categorized into two primary subdivisions: the lateral and medial septal nucleus. The lateral septum (LS) can be further delineated into distinct cytoarchitectural segments, namely, the dorsal, intermediate, and ventral components. The lateral septum is primarily composed of GABAergic neurons, which establish synaptic connections with several brain regions, including the ventral tegmental area and hypothalamus. Research has consistently shown that these regions play a significant role in regulating wakefulness and motivation (Luo et al., 2011; Vega-Quiroga et al., 2018). Optogenetic activation of the dorsal-intermediate lateral septum GABAergic neurons promoted arousal from sleep and accelerated emergence from anesthesia. Additionally, EEG burst activity showed an increase in number of bursts, a prolonged burst duration, and a decreased burst suppression ratio. Conversely, optogenetic inhibition of these neurons facilitated the shift from awake to non-rapid eye movement (NREM) sleep and extended the emergence time. The subsequent spectral analysis revealed an increase in the delta power and a concurrent decrease in the beta and gamma powers. But the burst activity did not cause a notable change (Wang et al., 2021a).

GABAergic neurons in the lateral septum directly innervate the ventral tegmental area (VTA) *via* a monosynaptic approach. Optogenetic activation of the dorsal–intermediate lateral septum GABAergic terminals in the ventral tegmental area shortened emergence time from isoflurane-induced general anesthesia, accompanied with a decreased delta power and increased beta power. On the contrary, optogenetic inhibition of GABAergic terminals in the ventral tegmental area prolonged the emergence time, with an increased delta power and a decreased theta power. Collectively, these findings indicate that the dorsal–intermediate lateral septum GABAergic neurons facilitate arousal which is at least partially mediated by projections to the ventral tegmental area (Wang et al., 2021a).

#### 5.2.2 Medial septum

The medial septum (MS) has been reported to play a pivotal role in arousal-based behaviors and the maintenance of wakefulness (Srividya et al., 2004; Leung et al., 2013; Fuhrmann et al., 2015; An et al., 2021). Chemogenetic inhibition of medial septum glutamatergic neurons increased the sensitivity, accelerated the induction of and prolonged the emergence from sevoflurane general anesthesia. Moreover, chemogenetic activation has the opposite effect. Optogenetic activation of the medial septum glutamatergic neurons is sufficient to promote cortical activation and behavioral emergence during steady-state of general anesthesia. Spectral analysis of EEG data showed that optogenetic activation of the medial septum glutamatergic neurons significantly reduced delta power, and increasing beta power and gamma power. Optogenetic activation of medial septal glutamatergic neurons elicited cortical activation and behavioral emergence during the burst-suppression state induced by deep sevoflurane anesthesia. Moreover, the burst suppression ratio decreased (Xia et al., 2024). By integrating *in vivo* fiber photometry recordings, genetic lesions, chemogenetic/optogenetic manipulations, EEG-EMG monitoring and behavioral tests, they revealed that the medial septum glutamatergic neurons exert an arousal-promoting role during various stages of sevoflurane anesthesia.

### 5.3 Other study of the basal forebrain

Several studies have found that cholinergic and glutamatergic neurons in the basal forebrain regulate the sleep-wake cycle (Deurveilher and Semba, 2011; Anaclet et al., 2015; Xu et al., 2015). To investigate the role of cholinergic and glutamatergic neurons in the basal forebrain during general anesthesia, Wang L et al. used optogenetics to selectively manipulate these neurons. Optogenetic activation of cholinergic neurons in the basal forebrain led to a prolonged induction time and a reduction in the emergence time. Similarly, optogenetic activation of glutamatergic neurons in the basal forebrain resulted in reduced sensitivity to propofol and a significantly longer induction time. Optogenetic stimulation-induced local field potential (LFP) oscillations showed an obvious increase in all frequency bands compared to pre-stimulation (Wang et al., 2021b). Besides, GABAergic neurons in the basal forebrain have been found participate in the regulation of general anesthesia. Activation of the basal forebrain GABAergic neurons with chemogenetics and optogenetics decreased sensitivity to isoflurane, prolonged the induction time, and accelerated emergence from isoflurane anesthesia. Optogenetic activation of the basal forebrain GABAergic neurons decreased delta power and the burst suppression ratio during 0.8% and 1.4% isoflurane anesthesia (Cai et al., 2023). In addition, chemogenetic activation of astrocytes in the basal forebrain had a significantly shortened isoflurane induction time, prolonged emergence time, and augmented delta power of EEG during anesthesia maintenance and recovery periods (Lin et al., 2023).

Furthermore, optogenetic activation of the basal forebrain GABAergic terminals in the thalamic reticular nucleus (TRN) also strongly promoted cortical activation and behavioral emergence from isoflurane anesthesia. EEG spectral analysis showed that optogenetic activation induced a notable decline in delta power and theta power, an increase in alpha power and beta power (Cai et al., 2023). Collectively, these results showed that the basal forebrain plays a pivotal role in facilitating behavioral and cortical emergence from general anesthesia.

## 6 Ventral tegmental area

The ventral tegmental area (VTA) is a midbrain region consisting of five subregions. It consists of a mixed population of neurons including dopaminergic, glutamatergic, and GABAergic neurons. (Morales and Margolis, 2017). As a primary hub for dopamine synthesis and release, the ventral tegmental area plays a crucial role in two distinct dopamine pathways. Specially, the limbic mesolimbic system pathway projects to the nucleus accumbens (NAc), while the mesocortical pathway projects to the frontal cortex (Gardner and Ashby, 2000).

### 6.1 Ventral tegmental area dopaminergic neurons

Dopaminergic neurons in the ventral tegmental area play a crucial role in many basic physiological and pathological functions, such as rewarding, addiction, motivation, emotion, reinforcement and cognition (Bouarab et al., 2019; Song et al., 2022). Although the ventral tegmental area dopaminergic neurons have been widely reported in regulating sleep-awake, the specific dopamine circuits in the brain that regulate sleep-awake are not clear. In addition, some studies have reported that the ventral tegmental area dopaminergic neurons participate in the induction and emergence of general anesthesia (Wang J. et al., 2023).

Optogenetic activation of the ventral tegmental area dopaminergic neurons induced a behavioral arousal response during continuous, steady-state general anesthesia with isoflurane. Spectral analysis of EEG recordings showed that optical stimulation resulted in significant decreases in delta power and alpha power. Pretreatment with the D1 receptor antagonist SCH-23390 before optogenetic activation of the ventral tegmental area inhibited the arousal responses and RORR. This study suggests that the ventral tegmental area dopaminergic neurons perform an important function in behavioral arousal and recovery of consciousness after general anesthesia (Taylor et al., 2016).

Next, there was a study showing that optogenetic activation of dopaminergic neurons in the ventral tegmental area extended the induction time and shortened the emergence time. Moreover, EEG recording showed an obvious difference during the induction period, including a decrease in delta bands, an increase in beta and gamma bands. Meanwhile, during the emergence period, theta bands decreased and beta bands and gamma bands increased (Gui et al., 2021). These results indicated that the hypnotic effect of general anesthetics, such as isoflurane and sevoflurane, may be attributed to the inhibition of the ventral tegmental area neurons (Leung, 2017).

In addition, chemogenetic activation of the ventral tegmental area dopaminergic neurons also prolonged the induction time and shortened the emergence time. Moreover, the spectral analysis of EEG showed that the delta band decreased while the beta band increased during the induction period. During the emergence period, the percentages of power in the delta and alpha bands decreased, while those in the theta, beta, and gamma bands increased (Song et al., 2022).

The nucleus accumbens receives dense dopaminergic projections from the ventral tegmental area. Chemogenetic and optogenetic activation of the ventral tegmental area dopaminergic terminals in the nucleus accumbens prolonged the induction time and reduced the emergence time. However, chemogenetic and optogenetic inhibition of these neurons have an opposite result. Moreover, during the induction and emergence periods, EEG analysis indicated that optical activation substantially declined the delta bands and increased the power of the gamma bands. Nevertheless, optogenetic inhibition increased the power of the delta bands and decreased the beta bands. These results demonstrate that the induction and emergence processes of sevoflurane anesthesia are regulated by the ventral tegmental area dopaminergic projections to the nucleus accumbens (Gui et al., 2021).

The medial prefrontal cortex (mPFC) is engaged in regulating the level of arousal. The prelimbic cortex (PrL) is a key region of the medial prefrontal cortex and receives dopaminergic projections from the ventral tegmental area. The results of chemogenetics combined with microinjection and optogenetics showed that activating the ventral tegmental area dopaminergic terminals in the prelimbic cortex pathway resulted in a prolonged induction time and shortened emergence time of sevoflurane anesthesia. EEG signal analysis during the induction period revealed that optogenetic activation of the ventral tegmental area dopaminergic terminals in the prelimbic cortex declined the percentages of power in the delta and alpha bands, increased in the gamma band. During the emergence period, the percentage of power in the delta band decreased, while the beta and gamma bands increased. These data indicated that the ventral tegmental area dopaminergic neurons promote emergence from anesthesia through projections to the prelimbic cortex (Song et al., 2022).

The olfactory tubercle (OT) is densely innervated by dopaminergic neurons from the ventral tegmental area as well, and the main neuronal components of the olfactory tubercle express D1-receptor and D2-receptor. Optogenetic activation of the ventral tegmental area dopaminergic terminals in the olfactory tubercle accelerates behavioral and cortical arousal but has no effect on the induction time of isoflurane anesthesia. In addition, EEG spectral analysis indicated a significant decreased in delta power, while the theta and alpha power increased during the emergence period. These results show that optical activation of dopaminergic terminals in the olfactory tubercle accelerates behavioral and cortical arousal (Yang et al., 2021).

### 6.2 Ventral tegmental area GABAergic neurons

GABAergic neurons in the ventral tegmental area are also involved in regulating sleep and wakefulness through their inhibitory projections to dentate gyrus (DG), lateral habenular nucleus (LHb), lateral preoptic area (LPO), and lateral hypothalamus (LH) (Yu et al., 2019). The firing rate of the ventral tegmental area GABAergic neurons is significantly reduced under general anesthesia. Optogenetic activation of the ventral tegmental area GABAergic neurons significantly increased the percentage of delta power, while reducing the percentage of beta and gamma waves. Conversely, optical inhibition of the ventral tegmental area GABAergic neurons significantly elevated the percentage of gamma power, while decreasing the percentage of delta, theta and alpha power. Chemogenetic activation of the ventral tegmental area GABAergic neurons led to a significant increase in the sensitivity to isoflurane anesthesia, which resulted in an accelerated induction time and prolonged emergence time. On the contrary, the chemogenetic inhibition of these neurons resulted in opposing effects on these time parameters (Yin et al., 2019).

Optogenetic activation of the ventral tegmental area GABAergic projections in the lateral hypothalamus shortened the induction time, and prolonged the emergence time, whereas optogenetic inhibition of these neurons prolonged the induction time. Moreover, EEG analysis showed that optogenetic activation of the ventral tegmental area GABAergic projections could significantly increase percentage of delta power and reduce percentage of gamma power during a light anesthesia state. Optogenetic inhibition of the ventral tegmental area GABAergic terminals in the lateral hypothalamus reduced the percentage of delta and alpha power and increased the percentage of gamma power. During a deep anesthesia with burst-suppression state, EEG recording confirmed that activation of the ventral tegmental area GABAergic neurons and projections in the lateral hypothalamus inhibited brain activity and led to an increase in BSR, whereas optogenetic inhibition of these neurons resulted in a decrease in BSR. These results indicate that the ventral tegmental area GABAergic neurons and their projections to the lateral hypothalamus are participated in the induction, maintenance and emergence of isoflurane anesthesia (Yin et al., 2019).

### 6.3 Ventral tegmental area glutamatergic neurons

Glutamatergic neurons in the ventral tegmental area mediate place preference (Wang et al., 2015), aversion (Root et al., 2014; Qi et al., 2016), innate defensive behaviors (Bouarab et al., 2019), and promote wakefulness (Yu et al., 2019). Optogenetic activation of the ventral tegmental area glutamatergic neurons prolonged the induction time and shortened the emergence time. Furthermore, EEG recording showed optogenetic activation of the ventral tegmental area glutamatergic neurons reduced the power percentage of delta band, increased the power percentage of beta and gamma bands. During the deep anesthesia, optogenetic activation of the ventral tegmental area glutamatergic neurons significantly reduced the BSR in EEG. However, optogenetic inhibition of the ventral tegmental area glutamatergic neurons has the opposite effect (Zhang S. et al., 2023). Furthermore, they proved that there were 26% of the ventral tegmental area glutamatergic neurons projected to the lateral septum (LS) GABAergic neurons. Optogenetic activation of the ventral tegmental area glutamatergic terminals in the lateral septum facilitates emergence from isoflurane anesthesia. optogenetic activation of the ventral tegmental area glutamatergic terminals in the lateral septum prolonged the induction time and shortened the emergence time. And EEG recording showed that optogenetic activation of the ventral tegmental area glutamatergic terminals in the lateral septum reduced the power percentage of delta band, increased the power percentage of gamma band during the light anesthesia, and a reduced BSR during the deep anesthesia. But optogenetic inhibition has the opposite effect (Zhang S. et al., 2023).

They did a comprehensive study of the role of VTA glutamatergic neurons during anesthesia and did work on neural circuits. And in the article, they pointed out the shortcomings of the study, which is worth learning and analyzing.

## 7 Thalamus

The thalamus, also known as the dorsal thalamus, is a nuclear complex situated in the diencephalon and is located on either side of the third ventricle (Herrero et al., 2002). The thalamus is the final subcortical structure that visual, auditory, and somatosensory information must travel through before reaching the cerebral cortex (McCormick and Bal, 1994).

### 7.1 Paraventricular thalamus (PVT)

The thalamus is the gate of the cerebral cortex, the ultimate target for the neural networks controlling behavioral states and cognitive functions. The paraventricular thalamus (PVT) in the paramedian thalamus has been widely researched due to its involvement in a number of behaviors, including fear conditioning, drug addiction, and feeding, all of which require elevated wakefulness (Ren S. et al., 2018). Ren et al. found that the paraventricular thalamus is a key control nucleus of wakefulness in the thalamus. In their experiments, they used optogenetic methods and found that optogenetic activation of the paraventricular thalamus glutamatergic neurons promoted arousal from isoflurane-induced general anesthesia. Similarly, optogenetic activation of the paraventricular thalamus glutamatergic neurons or their pathways occurred awake-like behavior from light isoflurane anesthesia and reduced the depth of anesthesia under deeper anesthesia (Ren S. et al., 2018). The experiments of Bu et al. found that chemogenetic activation of the paraventricular thalamus glutamatergic neurons resulted in accelerated emergence time but no effect on induction time in isoflurane anesthesia. In contrast, inhibition of the paraventricular thalamus glutamatergic neurons resulted in delayed emergence from isoflurane anesthesia without affecting anesthetic induction (Bu et al., 2022). Wang et al. found that chemogenetic activation of the paraventricular thalamus CR-expressing glutamatergic neurons reduced sensitivity to propofol, prolonged the induction time and decreased the emergence time. And chemogenetic inhibition has the opposite effect. Similarly, optogenetic inhibition of these neurons reduced the induced time and prolonged the emergence time during propofol anesthesia, and brief activation of these neurons using optogenetic stimulation induced fast and effective emergence at the cortical and behavioral levels under continuous steady-state propofol anesthesia (Wang Y.-L. et al., 2023). Duan et al. found that injection of esketamine (Esk) accelerated the emergence from isoflurane anesthesia in mice compared to saline injection. Chemogenetic inhibition of paraventricular thalamus glutamatergic neurons prolongs emergence from isoflurane anesthesia. Inhibiting the paraventricular thalamus glutamatergic neurons prolonged both induction time and emergence time after esketamine treatment (hM4Di-Esk vs. mCherry-Esk) and inhibition of the paraventricular thalamus glutamatergic neurons without esketamine showed a shorter induction time and a longer emergence time than with esketamine treatment (hM4Di-Saline vs. hM4Di-Esk) (Duan et al., 2024). These results are indicative of a role for paraventricular thalamus glutamatergic neurons in the transition of consciousness under anesthesia, but it is necessary to clarify the contribution of distinct neuronal pathways of the paraventricular thalamus.

Glutamatergic and GABAergic neurons of the paraventricular thalamus project to the bed nucleus of stria terminalis (BNST), which mediates starvation-induced arousal behavior (Hua et al., 2018). Chemogenetic inhibition of the paraventricular thalamus-bed nucleus of the stria terminalis pathway resulted a shortened induction time and a prolonged emergence time. Additionally, optogenetic activation of this pathway delayed the induction, accelerated the emergence, and induced behavioral arousal during continuous steady-state general anesthesia. Besides, it has been observed to decrease the level of anesthesia during burst suppression states. Furthermore, EEG recording showed that activation of these projections led to a rapid increase in burst duration and a significant increase in delta power compared with pre-stimulation period and a significantly decreased burst-suppression ratio. All these results indicate that the paraventricular thalamus glutamatergic neurons play a crucial role in regulating anesthesia states by their direct projections to the bed nucleus of the stria terminalis (Li et al., 2022).

### 7.2 Thalamic reticular nucleus (TRN)

The thalamic reticular nucleus (TRN) is a thin lamellar layer of cells between the outer medullary plate of the thalamus and the internal capsule that surrounds the anterior and lateral aspects of the main body of the thalamus, consists of a shell of GABAergic neurons, receives inputs from cortical and subcortical regions, has crucial roles in sensory processing, arousal and cognition (Pinault, 2004; McAlonan et al., 2006; Halassa et al., 2014; Dong et al., 2019). It has been involved in the regulation of anesthesia (Herrera et al., 2016; Zhang et al., 2019).

Optogenetic activation of the thalamic reticular nucleus rapidly induces local sleep-like thalamocortical slow waves, which reduce arousal states by decreasing thalamic firing. Awake animals exhibit less motor activity and an increased proportion of NREM sleep, anesthetized animals with reduced cortical activity exhibit dynamic changes tending to cortical shutdown (Lewis et al., 2015). However, Liu Y et al. found that optogenetic and chemogenetic activation of the thalamic reticular nucleus GABAergic neurons shortened the emergence time under propofol anesthesia without altering the induction time. On the contrary, optogenetic and chemogenetic inhibition of the thalamic reticular nucleus GABAergic neurons prolonged the emergence time. They showed that activation of anterior thalamic reticular nucleus GABAergic neurons facilitates wakefulness from propofol anesthesia (Liu C. et al., 2021).

### 7.3 Zona incerta (ZI)

The zona incerta (ZI) is a subthalamic nucleus located next to the reticular nucleus of the thalamus and is rich in GABA neurons, which form interconnections with many brain regions and are involved in the regulation of consciousness, sleep, and attention (Liu et al., 2015; 2017; Escudero and Nuñez, 2019). To explore the regulatory effects of GABAergic neurons in the zona incerta on sevoflurane and propofol anesthesia. Chemogenetic activation of the zona incerta GABAergic neurons accelerates induction in both sevoflurane and propofol anesthesia, but there was no difference in the emergence time. Optogenetic activation of the zona incerta GABAergic neurons showed the same effect. And EEG spectral showed optogenetic activation of the zona incerta GABAergic neurons has no significant effect on sevoflurane anesthesia maintenance. These results showed that GABAergic neurons in the zona incerta promote induction of anesthesia with sevoflurane and propofol, but do not modulate anesthesia maintenance and emergence (Cao et al., 2023). If inhibition of the zona incerta GABAergic neurons were added to this experiment it might be more complete and yield more reliable results.

## 8 Hypothalamus

### 8.1 Lateral hypothalamus (LH)

The lateral hypothalamus is vital in controlling the alteration of consciousness (Zhao et al., 2021a). There are two primary neuronal types in the lateral hypothalamus, including GABAergic neurons and glutamatergic neurons. Each type is divided into 15 subtypes. Specifically, there are 15 subtypes of glutamatergic neurons and 15 subtypes of GABAergic neurons (Mickelsen et al., 2019). For example, there is a group of glutamatergic neurons expressing orexin (hypocretin, Hcrt), which exerts a strong arousal-promoting effect. In addition, there is a group of glutaminergic neurons that express melanin-concentrating hormone (MCH), which has the opposite role to orexinergic neurons, and is active during sleep, especially during rapid eye movement (REM) sleep (Yang et al., 2022).

#### 8.1.1 Lateral hypothalamus glutamatergic neurons

Glutamatergic neurons have a large population in the lateral hypothalamus and send dense projections towards the lateral habenula (LHb) (Zhao et al., 2021a). The lateral hypothalamus glutamatergic neurons are thought to regulate sleep state (Alam and Mallick, 2008; John et al., 2008). Chemogenetic activation of the lateral hypothalamus glutamatergic neurons noticeably prolonged induction time and recovery from an isoflurane-induced general anesthesia was accelerated. In contrast, chemogenetic inhibition of these neurons shortened the induction time and prolonged the emergence time (Zhao et al., 2021a). Similarly, chemogenetic manipulation of such neurons under propofol anesthesia demonstrated this. Huang et al. found that chemogenetic inhibition of glutamatergic neurons in the lateral hypothalamus facilitated induction and prolongs emergence from propofol anesthesia. In contrast, chemogenetic activation of these neurons increased the induction time (Huang et al., 2024). Optical activation of glutamatergic neurons in the lateral hypothalamus reduces the depth of isoflurane anesthesia. During a burst-suppression state, optogenetic activation resulted in a significant decline of BSR. And during a light anesthesia, optogenetic activation of the lateral hypothalamus glutamatergic neurons decreased the total power percentage of the delta waves, but increased the power percentage of alpha waves, beta waves and gamma waves. Optogenetic inhibition of the lateral hypothalamus glutamatergic neurons only induced a decreased beta waves during the light anesthesia (Zhao et al., 2021a).

Furthermore, optogenetic activation of the lateral hypothalamus glutamatergic terminals in the lateral habenula (LHb) promoted emergence from anesthesia and shifted the depth of anesthesia to a lighter level as well. Optogenetic activation of the lateral hypothalamus glutamatergic terminals in the lateral habenula prolonged the induction time, reduced the emergence time. During a deep anesthesia with burst-suppression state, optogenetic activation of lateral hypothalamus glutamatergic terminals in the lateral habenula reduced BSR. Under a lighter anesthesia, optogenetic activation of the lateral hypothalamus glutamatergic terminals in the lateral habenula decreased the power percentage of the delta waves, increased power percentages of the beta waves and the gamma waves. On the contrary, optogenetic inhibition of the lateral hypothalamus glutamatergic terminals in the lateral habenula induced the opposite effect. Optogenetic inhibition of the lateral hypothalamus glutamatergic terminals in the lateral habenula reduced the induction time, prolonged the emergence time. During a light anesthesia state, optical inhibition of the lateral hypothalamus glutamatergic terminals in the lateral habenula increased the power percentage of the delta waves, reduced power percentages of the beta waves and the gamma waves (Zhao et al., 2021a). Therefore, the lateral hypothalamus glutaminergic neurons and projections in the lateral habenula are essential for both the induction and emergence of anesthesia.

#### 8.1.2 Lateral hypothalamus orexinergic neurons

A number of experiments have shown that the orexin is involved in arousal from general anesthesia. Optogenetic activation of orexinergic neurons in the lateral hypothalamus can not only facilitated the emergence time after deep isoflurane anesthesia, but also directly promoted recovery from light isoflurane anesthesia (Xiang et al., 2022).

Furthermore, optogenetic activation of orexinergic neuron terminals in the basal forebrain and locus coeruleus decreased the emergence time. This suggests that activation of orexinergic terminals in these brain regions plays a role in facilitating emergence from anesthesia. Specifically, optogenetic activation of orexinergic terminals in the locus coeruleus reduced BSR under deep isoflurane anesthesia. Conversely, during a light isoflurane anesthesia, the delta power decreased, whereas the power of the beta and gamma bands increased. Moreover, optical stimulation increased behavioral movements in ChR2-mCherry rats and induced the righting response. Orexinergic terminals in the basal forebrain and the locus coeruleus were not involved in the induction process of isoflurane anesthesia (Wang L. et al., 2021).

Optogenetic activation of the perifornical area of the lateral hypothalamus (PeFLH) orexinergic neurons reduced BSR in the burst-suppression state induced by desflurane and isoflurane anesthesia, but optogenetic inhibition had no effect on BSR. Moreover, optogenetic activation of orexinergic neuron terminals in the paraventricular thalamus also caused a decrease in BSR. It indicates that optogenetic activation of the perifornical area of the lateral hypothalamus orexinergic neurons and its terminals in the paraventricular thalamus reduces the depth of anesthesia with desflurane and isoflurane (Zhao et al., 2021b).

Chemogenetic activation of the orexinergic projections to the lateral habenula (LHb) shortened the emergence time, while the inhibition of this projection prolonged the time of emergence. optical activation of the orexinergic terminals in the lateral habenula significantly decreased BSR, and optogenetic inhibition has the opposite effect. Spectral analysis of the EEG revealed that acute optical stimulation induced a significant decrease in delta power, theta power, and alpha power. Collectively, these findings indicated that activation of the lateral habenula orexinergic terminals was sufficient to induce cortical activation and behavioral emergence in mice from the light anesthesia state (Zhou et al., 2023).

#### 8.1.3 Lateral hypothalamus GABAergic neurons

In addition to glutamatergic and orexinergic neurons, GABAergic neurons in the lateral hypothalamus participate in the regulation of sleep and wakefulness as well. GABAergic neurons of the lateral hypothalamus exert a substantial inhibitory control over the thalamic reticular nucleus GABAergic neurons. Optogenetic activation of the lateral hypothalamus GABAergic terminals in the thalamic reticular nucleus during an isoflurane-induced burst-suppression state significantly prolonged the total duration of burst activity in cortex. In some cases, optical stimulation during bursting led to emergence from anesthesia accompanied by limb movements and the recovery of righting reflex. These results further implicate the thalamic reticular nucleus in regulating the lateral hypothalamus GABAergic neurons activation of the thalamocortical system (Herrera et al., 2016).

### 8.2 Paraventricular hypothalamic nucleus (PVH)

The paraventricular hypothalamic nucleus (PVH) is located ventral to the mesencephalon, adjacent to the third ventricle, and has dense reciprocal projections to structures that regulate sleep-wake behavior and anesthesia (Kaur et al., 2013; Du et al., 2018; Wang et al., 2019; Hayat et al., 2020; Chen et al., 2021; Wang X. et al., 2021). Chemogenetic activation of the paraventricular hypothalamic nucleus glutamatergic neurons prolonged induction time and shortened emergence time from anesthesia by decreasing the depth of anesthesia during 1% and 4% isoflurane anesthesia. However, chemogenetic inhibition of the paraventricular hypothalamic nucleus glutamatergic neurons facilitated the induction process and delayed the emergence accompanied by deepening the depth of anesthesia. This study suggests that the paraventricular hypothalamic nucleus glutamatergic neurons are significant in preventing narcosis and promoting arousal from isoflurane anesthesia (Yin et al., 2023).

### 8.3 Preoptic area (POA)

The preoptic area (POA) has been shown to modulate arousal in both natural (sleep and wake) and drug-induced (anesthetic-induced unconsciousness) states. The preoptic area is anatomically separated into four regions: the medial preoptic area (MPO), the lateral preoptic area (LPO), the median preoptic area (MnPO) and the ventrolateral preoptic area (VLPO). High densities of sleep-active neurons are located primarily in the ventrolateral preoptic area and the median preoptic area (Reitz and Kelz, 2021). Studies have shown that chemogenetic and optogenetic activation of the ventrolateral preoptic area galaninergic neurons significantly increased NREM sleep (Kroeger et al., 2018). However, chemogenetic activation of the medial preoptic area and the ventrolateral preoptic area GABAergic neurons altered sleep-wake architecture without affecting anesthetic sensitivity or emergence time (Vanini et al., 2020). So these results suggest that the median preoptic area and the ventrolateral preoptic area, while altering the sleep-wake cycle, cannot be shown to influence the anesthesia process.

### 8.4 Hypothalamic tuberomammillary nucleus (TMN)

The hypothalamic tuberomammillary nucleus (TMN) has been suggested to play a crucial role in the sedative response to GABAergic anesthetics (Nelson et al., 2002). However, some studies have found the TMN GABAergic neurons to be associated with the control of wakefulness (Yu et al., 2015) but others hold the opposite view (Venner et al., 2019). To further investigate this matter, recent research conducted by Liu et al. employed chemogenetics and optogenetics. Firstly, chemogenetic stimulation of the hypothalamic tuberomammillary nucleus GABAergic neurons resulted in decreased sensitivity to sevoflurane, yet chemogenetic inhibition enhanced the sensitivity. In the activation group, the induction time was prolonged and the emergence time was shortened, whereas in the inhibition group, the induction time was shortened. Secondly, during propofol anesthesia, chemogenetic activation of these neurons significantly also increased anesthetic sensitivity and significantly shortened the emergence time. But there was no significance in the inhibition group. Optogenetic activation of these neurons produced the same results. And EEG recording indicated a reduced anesthesia depth. These results showed that activation of GABAergic neurons in the hypothalamic tuberomammillary nucleus attenuates the anesthetic effects of sevoflurane and propofol (Liu et al., 2023).

## 9 Other brain areas

### 9.1 Parabrachial nucleus (PBN)

The parabrachial nucleus (PBN), a pivotal brain region that is closely associated with arousal (Kaur et al., 2013), possesses a vast network of glutamatergic neurons that project to multiple arousal-enhancing areas in the brain. These areas include the basal forebrain, lateral hypothalamus, thalamus, amygdala complex, and cortex (Saper and Loewy, 1980; Saper, 1982; Fulwiler and Saper, 1984; Herbert et al., 1990), which are all well-documented players in the process of general anesthesia (Wang et al., 2019). Chemogenetic activation of the parabrachial nucleus glutamatergic neurons had a significant effect on sevoflurane anesthesia, increasing the ED50 for LORR, prolonging induction time, and reducing emergence time. Instantaneous optogenetic activation of the parabrachial nucleus glutamatergic neurons can produce cortical arousal. On the contrary, chemogenetic inhibition of these neurons had the opposite effect, slightly prolonged emergence time. This study suggested that the parabrachial nucleus glutamatergic neurons is efficient to regulate the state of sevoflurane-induced anesthesia (Wang et al., 2019).

The function of the parabrachial nucleus in projecting to other brain regions has also been investigated. Firstly, optogenetic activation of the parabrachial nucleus terminals in the lateral hypothalamus notably decreased the emergence time, while there was no notable difference in the induction time. And the BSR obviously decreased. Optogenetic inhibition of the parabrachial nucleus terminals in the lateral hypothalamus delayed emergence from isoflurane anesthesia and no difference in emergence time. And there was no influence in the BSR. Optogenetics activation or inhibition of the parabrachial nucleus terminals in the basal forebrain got the same results in induction time, emergence time and BSR as the parabrachial nucleus terminals in the lateral hypothalamus (Lu et al., 2023). Inspired by Qiu et al.'s chemogenetics study of these two pathways (Qiu et al., 2016), they did an optogenetic study in the field of anesthesiology, which yielded the same results demonstrating the important role of the parabrachial nucleus projections in the emergence of isoflurane anesthesia.

### 9.2 Lateral habenula (LHb)

The habenula is located in the posterior part of the thalamus above the midline and can be divided into two regions: the medial habenula (MHb) and the lateral habenula (LHb). The lateral habenula plays a role primarily in reward processing, stress adaptation, sleep and circadian rhythm regulation (Liu C. et al., 2021). Chemogenetic activation of the lateral habenula glutamatergic neurons shortened the induction time, prolonged the emergence time and increased BSR associated with isoflurane anesthesia. On the contrary, chemogenetic inhibition of the lateral habenula glutamatergic neurons induced a longer induction time, and led to a shorter emergence time. Moreover, optogenetic activation of the lateral habenula glutamatergic neurons accelerated the induction process with an increase in delta waves and a decrease in bata and gamma waves, and delayed the emergence time with a complementary increase of delta band and a decrease in gamma band (Liu Y. et al., 2021).

Optogenetic activation of the lateral habenula glutamatergic axonal terminals in the rostromedial tegmental nucleus (RMTg) resulted a reduced induction time accompanied by an increase of delta wave and a decrease in alpha, beta and gamma waves. On the other hand, it resulted a prolonged emergence time with the augment of total power percentages of delta waves and the decrease of total power percentages of beta and gamma waves. These results suggest that the lateral habenula glutamatergic neurons or projections to the rostromedial tegmental nucleus is capable of modifying modulate the anesthesia state of isoflurane (Liu Y. et al., 2021).

### 9.3 Ventrolateral periaqueductal gray (vlPAG)

The periaqueductal gray (PAG), a structure that surrounds the brain’s aqueducts, plays a critical role in the regulation of pain, the autonomic nervous system, defense responses, depressive behaviors, and sleep (Weber et al., 2018; Silva and McNaughton, 2019; Zhong et al., 2019). And GABAergic neurons in the ventrolateral periaqueductal gray (vlPAG) is important for regulating sleep state transitions (Zhong et al., 2019). Optogenetic activation of the ventrolateral periaqueductal gray (vlPAG) GABAergic neurons shortened the emergence time. EEG recording and behavioral tests also showed that optogenetic activation promoted recovery from sevoflurane anesthesia. While inhibiting these neurons delayed the emergence process. Chemogenetic activation of the ventrolateral periaqueductal gray shortened the emergence time, while inhibition of these neurons prolonged the emergence time (Guo et al., 2023).

Optogenetic activation of the ventrolateral periaqueductal gray GABAergic projections in the ventral tegmental area facilitated arousal, as evidenced by a shortened emergence time and EEG recording changes. However, optogenetic activation of GABAergic terminals in the lateral preoptic area (LPO) and zona incerta (ZI) had no significant effect on sevoflurane anesthesia (Guo et al., 2023).

The downstream projection to the periaqueductal gray is mainly from the medial prefrontal cortex (mPFC) (Hardy and Leichnetz, 1981). Optogenetic activation of the medial prefrontal cortex projections to the ventrolateral periaqueductal gray facilitates arousal from sevoflurane anesthesia (Guo et al., 2023). These results indicate that ventrolateral periaqueductal gray GABAergic neurons promote arousal of sevoflurane anesthesia through cortico-midbrain circuit.

### 9.4 Dorsal raphe nucleus (DRN)

The dorsal raphe nucleus, located in the midbrain, is primarily populated by serotonin (5-HT) neuron, and closely associated with psychiatric disorders such as pleasure deficit, anxiety and depression (Ren J. et al., 2018). Optogenetic activation of the dorsal raphe nucleus serotonergic neurons notably reduced BSR, whereas optogenetic inhibition of the dorsal raphe nucleus serotonergic neurons increased BSR during anesthesia maintenance. Furthermore, chemogenetic activation of the dorsal raphe nucleus serotonergic neurons shortened the emergence time. On the contrary, chemogenetic inhibition of the dorsal raphe nucleus serotonergic neurons prolonged the emergence time. In conclusion, activation of the dorsal raphe nucleus serotonergic neurons could reduce the depth of anesthesia and promote emergence from isoflurane anesthesia (Li et al., 2021). Ma et al. found although optogenetic activation of the dorsal raphe nucleus serotonergic neurons showed little influence on the induction time, the emergence time was significantly shortened (Ma et al., 2023). And they did extensive and detailed research on the serotonergic neuron, which affects the emergence time of sevoflurane anesthesia. In summary, both of these studies showed that the dorsal raphe nucleus serotonergic neurons can shorten the emergence time.

### 9.5 Locus coeruleus (LC)

The locus coeruleus (LC), a brainstem pontine nucleus of noradrenergic neurons, sends widespread outputs to many brain regions to regulate a variety of functions, including attention,sleep-wake states, and the general anesthetic state (Ao et al., 2021). Chemogenetic activation of the locus coeruleus tyrosine-hydroxylase (TH) neurons did not noticeably alter the time of induction, but rather reduced the emergence time. Additionally, the locus coeruleus tyrosine-hydroxylase neurons activation promoted cortical arousal, as evidenced by changes in EEG activity. Specifically, there was an obvious decrease of delta power and increase of alpha power during a light anesthesia, although no BSR changes were evident under a deep anesthesia (Ao et al., 2021). These findings indicate that the locus coeruleus plays an important role in modulating emergence from anesthesia, without significantly affecting the induction process.

The paraventricular thalamus receives dense tyrosine-hydroxylase inputs from the locus coeruleus. It was found that chemogenetic activation of the locus coeruleus tyrosine-hydroxylase neurons resulting in enhanced c-Fos expression in the paraventricular thalamus, which indicates that the locus coeruleus tyrosine-hydroxylase neurons project to the paraventricular thalamus. Furthermore, optogenetic activation of the locus coeruleus tyrosine-hydroxylase fibers in the paraventricular thalamus produces electrophysiological evidence of arousal and facilitated emergence from anesthesia, whereas chemogenetic inhibition of the locus coeruleus tyrosine-hydroxylase projections in the paraventricular thalamus prolonged the emergence time of anesthesia (Ao et al., 2021). These findings indicate that the locus coeruleus - the paraventricular thalamus pathway is engaged in emergence from anesthesia.

### 9.6 Rostromedial tegmental nucleus (RMTg)

The rostromedial tegmental nucleus (RMTg) is a distinct mesopontine GABAergic structure that stretches from the caudal pole of the ventral tegmental area deep into the mesopontine tegmentum (Zhao et al., 2020). GABAergic neurons in the rostromedial tegmental nucleus send inhibitory projections to multiple arousal-promoting nuclei (Vlasov et al., 2021). Furthermore, studies found a novel role for the rostromedial tegmental nucleus regulating sleep–wake behavior (Yang et al., 2018). In addition, chemogenetic activation of GABAergic neurons in the rostromedial tegmental nucleus produced a NREM sleep-like state with enhanced sensitivity to sevoflurane-induced unconsciousness (Vlasov et al., 2021).

### 9.7 Pedunculopontine tegmental nucleus (PPT)

The pedunculopontine tegmental nucleus (PPT), located within the pontine tegmentum, contains a diverse population of glutamatergic, cholinergic, and GABA neurons which are involved in a variety of processes including arousal, consciousness, cognition, sleep-wake cycles, and sensory integration (Vitale et al., 2019). The distinct types of the pedunculopontine tegmental nucleus glutamatergic neurons have varying impacts on sleep/wake behavior, and only the activation of the pedunculopontine tegmental nucleus glutamatergic neurons can trigger prolonged cortical activation and behavioral arousal (Kroeger et al., 2017). Chemogenetic inhibition of the pedunculopontine tegmental nucleus glutamatergic neurons accelerated induction of anesthesia, delayed emergence from sevoflurane anesthesia, and increased sevoflurane sensitivity. Optogenetic activation of the pedunculopontine tegmental nucleus glutamatergic neurons drove cortical activation and behavioral arousal during steady-state sevoflurane anesthesia. And the EEG spectral analysis showed a rapid transition from an anesthesia state (slow-wave activity) to an awake state (low-voltage fast activity) and reduced the BSR during burst suppression state (Li et al., 2024).

During steady-state anesthesia with sevoflurane, pedunculopontine tegmental nucleus-ventral tegmental area circuit activation induces cortical activation and behavioral arousal. EEG recordings show that optogenetic activation of pedunculopontine tegmental nucleus glutamatergic terminals in the ventral tegmental area caused a rapid transition from anesthesia to wakefulness and a decrease in BSR (Li et al., 2024). These results indicated that the pedunculopontine tegmental nucleus glutamatergic neurons are associated with induction and arousal of anesthesia.

## 10 Perspectives

In this article, we review studies that have applied chemogenetics and optogenetics to manipulate neural circuits under general anesthesia. For many years, traditional techniques including electrical stimulation, microinjection, and microdialysis were used for the mechanism of anesthesia research. These methods, however, cannot be used to activate or inhibit specific populations of neurons, because different types of cells in the targeted brain region will be affected indiscriminately (Melonakos et al., 2020). Using optogenetics and chemogenetics reviewed here, scientists have discovered that many nuclei play essential regulatory roles in general anesthesia induced unconsciousness.

Using optogenetics and chemogenetics further advanced our understanding in the neural mechanism of general anesthesia induced unconsciousness. A few conclusions can be drawn from all the reviewed investigations. 1) These studies further support the theory that general anesthetics produce unconsciousness by targeting numerous neural circuits in cortical and subcortical regions. 2) Most of the studies on the neural mechanisms of general anesthesia have been inspired by the neural circuits of sleep-wakefulness regulation. 3) Chemogenetic or optogenetic activating arousal nuclei prolongs anesthesia induction time, accelerates emergence or facilitates EEG activation. Restores consciousness in anesthetized subjects. On the contrary, activation of arousal-inhibiting neurons shorten anesthesia induction time and delay emergence. 4) Most neural circuits involved in sleep-arousal regulation also play a role in anesthesia-induced loss and recovery of consciousness. However, the neural circuit for sleep and general anesthesia may not completely overlap. 5) The molecular targets of anesthesia induced unconscious and emergence may not share an identical mechanism. 6) Different anesthetics may not share the same neural circuits. The most frequently studied anesthetics were isoflurane and sevoflurane.

However, each technique has its advantages and disadvantages. It is suggested to consider the technical limitation of chemogenetic or optogenetic when interpreting experimental results. For optogenetics, firstly, fiber optic implantation may damage brain tissue, particularly if deep brain regions or multiple brain regions need to be stimulated at the same time, the damage to brain tissue is greater, which may affect its normal physiological function (Ung and Arenkiel, 2012). The second limitation is that the light is partially lost as it passes through the fiber and brain tissue, thus affecting its efficiency and potentially affecting the judgment of experimental results. In addition, viral transfection may be less than satisfactory, which also has a large impact on its efficiency. Ultimately, the interpretation of negative behavioral outcomes may be confounded by these factors. In addition, long-term high-energy optical stimulation may produce thermal effects, triggering physiological responses that impede the accurate interpretation of experimental findings (Carter et al., 2010).

Chemogenetics also has some significant disadvantages. Firstly, despite CNO being categorized as an inert ligand, it undergoes metabolic conversion to the antipsychotic medication clozapine. Given that clozapine has a sedative effect, employing high doses of CNO (>5 mg/kg) could potentially impact the interpretation of experimental outcomes (Gomez et al., 2017). As a result, the current prevalent systemic dosage of CNO is constrained to a range of 0.6–3 mg/kg. Secondly, CNO affects all neurons expressing chemoreceptors when administered systemically. Unlike optogenetics, which can precisely target specific brain regions through optical fiber implantation, chemogenetics necessitates the regulation of receptor expression range through meticulous viral injections with low volumes. Thirdly, chemogenetics exhibits comparatively less temporal precision in comparison to optogenetics. The peak effect of CNO occurs within the window of 30–60 min after administration and endures for approximately 9 h (Alexander et al., 2009).

Future research may focus on the specific effects of various anesthetics, especially for intravenous anesthetics on the same neural nuclei or circuits. Except for dopaminergic, glutamatergic and cholinergic neurons, many other types neurons have not yet been extensively reported and should be the focus of future research. Patch-clamp combined with optogenetics is a new method for studying complex network connectivity in brain regions-cortex under general anesthesia. It allows simultaneous layer recordings and targeted patch-clamp recordings of selected neuronal populations. With this approach, it is possible not only to probe discrete components of complex networks, but also to elucidate potential mechanisms by which anesthetics influence evoked responses (Murphy et al., 2021).

In summary, application of chemogenetics and optogenetics advanced our understanding of general anesthesia not only at a specific nuclei, but also the type of neurons as well as potential neural circuits modulated by anesthetics. Hopefully, with the progress in new technology, the understanding the mechanism of anesthesia induced consciousness will continue to advance.
